# A Multidimensional Analysis of Shade Selection Difficulty for Indirect Restorations Among Dental Students and Professionals

**DOI:** 10.3390/dj14040234

**Published:** 2026-04-14

**Authors:** Roxana-Ionela Vasluianu, Andreas Katsonis, Monica Silvia Tatarciuc, Anca Mihaela Vitalariu, Adina Oana Armencia, Andrea-Simoni Katsoni, Panagiotis Perperidis, Catalina Cioloca Holban, Irina Gradinaru, Ovidiu Stamatin, Magda Ecaterina Antohe

**Affiliations:** 1 Grigore T. Popa University of Medicine and Pharmacy, 700115 Iasi, Romania; roxana.vasluianu@umfiasi.ro (R.-I.V.); md-eng-10374@students.umfiasi.ro (A.K.); tatarciucm@yahoo.com (M.S.T.); anca.vitalariu@umfiasi.ro (A.M.V.); catalinaholban2906@gmail.com (C.C.H.); irina.gradinaru@umfiasi.ro (I.G.); ovidiu.stamatin@umfiasi.ro (O.S.); magda.antohe@umfiasi.ro (M.E.A.); 2 Oral Diagnosis and Radiology, National and Kapodistrian University of Athens, 11527 Athens, Greece; skatsoni@uoa.gr; 3 Private Clinic, 10552 Athens, Greece; perperidis2001@gmail.com

**Keywords:** shade selection, polychromatic teeth, dental students, human perception, technology, dental shade matching, cognitive load, educational protocols

## Abstract

Despite advances in dental materials and digital color registration systems, esthetic matching remains a clinical challenge for both dental students and experienced professionals. A comprehensive narrative review was conducted through bibliographic searches in PubMed, Scopus, Web of Science, and PsycINFO databases from January 2015 to January 2026. The evidence was synthesized using a four-dimensional analytical framework encompassing technological, cognitive–psychological, educational, and clinical-contextual factors. Quantitative synthesis revealed substantial variability in shade matching success rates, with intraoral scanners demonstrating pass rates ranging from 31.3% to 78.2% across devices, while spectrophotometers achieved superior repeatability (ICC > 0.9) but faced interpretive barriers. Cognitive load theory explains the performance deterioration, with novices being particularly susceptible to retinal fatigue and metamerism under non-standardized lighting conditions. The proposed paradigm shift involves redefining shade selection from a purely technical task to a cognitive skill that requires deliberate perceptual calibration, structured educational protocols, and hybrid digital visual workflows. To improve esthetic predictability, educational programs need to integrate longitudinal training in color science with objective feedback mechanisms. Clinical workflows should adopt hybrid calibration-centric protocols that position technology as a verification tool, rather than a replacement for clinical judgment. Understanding the multidimensional nature of shade matching difficulty enables the development of evidence-based educational protocols and clinical workflows, ultimately improving esthetic outcomes.

## 1. Introduction

The clinical importance of accurate shade matching is underscored by remake rate data. Systematic reviews indicate that esthetic dissatisfaction accounts for 18–32% of all indirect restoration remakes, with shade mismatch cited as the primary reason for patient rejection in approximately 25% of anterior restorations [1,2]. Among dental laboratories, up to 40% of returned cases involve shade-related discrepancies, representing substantial economic costs (estimated at $200–500 per remake) and chair time inefficiencies [3]. These figures persist despite technological advances, highlighting the refractory nature of this clinical challenge.

The present review organizes the evidence according to four interconnected dimensions:

1. The cognitive–psychological dimension encompasses visual perception limitations, color memory decay, retinal fatigue, metamerism, and cognitive load theory, factors that constrain the clinician’s ability to accurately perceive and discriminate color independently of technology.
2. The technological dimension addresses the performance characteristics, variability, and usability limitations of spectrophotometers, intraoral scanners, and digital shade matching systems, including inter-device agreement and the interpretive burden of numerical outputs.
3. The educational dimension examines how shade selection is taught, practiced, and assessed in dental curricula, including the simulation–clinic gap, feedback mechanisms, and developmental trajectories from novice to expert.
4. The clinical-contextual dimension incorporates environmental factors (lighting, operatory setup), biological dynamics (tooth dehydration, soft-tissue reflectance), patient-specific variables, and clinician-technician communication pathways.

For the purposes of this review, ‘indirect restorations’ refer exclusively to laboratory-fabricated or milled restorations (crowns, veneers, inlays, onlays, and fixed partial dentures) that are fabricated ex vivo and subsequently cemented. Semi-direct techniques, including chairside CAD/CAM composite restorations, are excluded due to fundamental differences in material optical properties and shade selection workflows.

Achieving a discreet restoration that blends harmoniously with existing natural teeth is fundamental to contemporary cosmetic dentistry. The current era signifies a phase of unparalleled technical progress. Spectrophotometers that quantify color with exceptional precision, intraoral scanners that capture three-dimensional topography and color data, and advanced material systems that reproduce the optical complexity of natural dentition are increasingly accessible [4,5]. What is the reason for this paradox?

The answer lies not in the technology itself, but in the complex human–technology interface and the fundamentally multifaceted nature of collaborations. Hein et al. have recently demonstrated that, even when using identical devices under standardized conditions, shade matching performance varies dramatically, with clinical success rates ranging from 31.3% to 78.2% between different intraoral scanners [6]. This variability cannot be attributed solely to device limitations, but rather reflects the complex interplay between technological capability, human perception, cognitive processing, educational training, and clinical context.

The clinical context is a domain where theoretical knowledge, perceptual limitations, and technological precision intersect with biological variability and environmental uncertainty. Color selection in this context does not arise exclusively from isolated technical errors, but rather from the non-standardized operating environment, the dynamic optical properties of dental tissues, the complex interactions between the clinician, the patient and the materials, and the deficiencies in communication between the clinician and the technician [7,8,9].

This analysis aims to highlight the factors that contribute to and, at the same time, influence color selection throughout the entire professional development process, from student to expert. The data from the literature were structured in a four-dimensional framework that includes clinical-contextual, technological, cognitive–psychological and educational aspects. By understanding the interaction and evolution of these dimensions observed through experience, simplistic solutions can be overcome, and strategies can be developed to address the unique challenges faced by each phase of professional development.

This review advances beyond previous contributions in three respects. First, while prior systematic reviews have focused on comparative accuracy of specific devices or methods [10,11], the present analysis adopts a multidimensional framework that explicitly maps interactions between cognitive, technological, educational, and contextual factors. Second, this review integrates findings from cognitive psychology and educational science, disciplines traditionally absent from dental color science, to explain why performance variability persists despite technological standardization. Third, we propose a calibration-centric clinical protocol and evidence-based educational recommendations derived explicitly from the multidimensional analysis, bridging the gap between descriptive synthesis and prescriptive guidance.

## 2. Materials and Methods

### 2.1. Search Strategy

A literature search was conducted in four electronic databases: PubMed (including MEDLINE), Scopus, Web of Science Core Collection, and PsycINFO. The search period extended from 1 January 2015, to 31 January 2026. This 11-year window was selected to encompass the period after the widespread clinical adoption of intraoral scanners with integrated color capture.

Equivalent search strings were adapted for the syntax requirements of Scopus, Web of Science, and PsycINFO. Two independent reviewers (authors) performed the initial search and screened the titles and abstracts. Disagreements were resolved by discussion or consultation with a third reviewer. Duplicate records were identified using manual deduplication.

### 2.2. Inclusion and Exclusion Criteria

Studies were included if they met the following PICOS-derived criteria:

○ Population: Dental students (any year of study), dental professionals (general practitioners, prosthodontists, restorative specialists, dental technicians), or comparison groups that included both;
○ Intervention: Shade matching or shade selection for indirect restorations using any method (visual, spectrophotometric, scanner-based, photographic);
○ Comparator: Alternative shade matching method, reference standard (spectrophotometric ΔE values), or different operator groups;
○ Outcomes: Quantitative measures of shade matching accuracy (e.g., hit/reject rates, ΔE values, percentage of correction), agreement statistics (kappa, ICC), or qualitative assessments of perceived difficulty or confidence;
○ Study types: Original research (randomized controlled trials, controlled clinical trials, cross-sectional studies, cohort studies, case–control studies, diagnostic accuracy studies) and systematic reviews with meta-analyses.

Additional inclusion criteria: English language; human subjects; publication date 2015–2026; full text availability.

Exclusion criteria: Studies focused exclusively on direct composite restorations; semi-direct CAD/CAM composite restorations; temporary dentition; and in vitro studies using extracted teeth without clinical simulation.

### 2.3. Study Quality Assessment

The methodological quality of the included studies was assessed using custom-designed tools based on study design. For cross-sectional and observational studies, the AXIS (Appraisal tool for Cross-Sectional Studies) tool was used, assessing 20 items based on study design, sample size justification, risk of bias, and completeness of reporting. For diagnostic accuracy studies (comparison of shade matching methods to reference standards), the QUADAS-2 (Quality Assessment of Diagnostic Accuracy Studies) tool was applied. Given the narrative synthesis approach of this review, studies were not excluded based on quality scores. However, the quality assessments informed the strength of the findings and are reported in the discussion of limitations.

### 2.4. Data Extraction and Synthesis

Data were extracted independently by two reviewers using a standardized form that included: first author, year, study design, population characteristics (sample size, education/experience level), shade matching method(s), reference standard, outcome measures, and main findings. Discrepancies were resolved by consensus. Evidence was synthesized using the four-dimensional framework described in the Introduction, with a thematic analysis that grouped findings into cognitive–psychological, technological, educational, and clinical-contextual domains.

## 3. The Cognitive Psychological Dimension

### 3.1. Visual Perception, Metamerism and Cognitive Load Theory

Shade selection is fundamentally constrained by the physiological and perceptual constraints of the human visual system. Numerous studies have shown that visual shade matching is not always accurate or consistent. This is because lighting, observer fatigue, and context can alter the results [12]. Human color perception is highly dynamic and context dependent, with surrounding structures such as the gingiva, lips, and adjacent teeth all potentially altering the perceived hue, chroma, and value of a tooth, leading to systematic perceptual errors [13,14,15].

Impaired color memory further complicates the process. Because precise color memory fades rapidly, clinicians must constantly compare shade palettes to natural dentition, making memorization more difficult and reproduction less likely [12]. Retinal fatigue caused by bright operating lights makes cones less responsive, making it more difficult to differentiate colors over time [16,17]. Metamerism, where two colors look the same under one light but different under another, further exacerbates these perceptual limitations. Research consistently demonstrates that non-standardized lighting significantly increases shade matching errors [18,19,20].

In 2024, Jouhar et al. showed that dental students were significantly better at shade matching under corrective lighting than under clinical fluorescent lighting. The students’ accuracy was statistically related to the lighting conditions. This finding underscores the essential function of standardized lighting in facilitating accurate visual assessment [13]. These results are supported by other studies that show that dental students often have too much information to process when trying to match shades, making them less accurate and more likely to use simple strategies [12,21].

Choosing a shade places a lot of stress on the brain, as clinicians must simultaneously assimilate a lot of visual, contextual, and material related information. This is consistent with the Cognitive Load Theory, which states that performance deteriorates when the demands of a task are too great for working memory to handle [22].

In shade selection:

○ Intrinsic task arises from the fact that teeth have complex optical properties, natural dentition has a variety of colors, translucency varies, and color is spread in three dimensions [23,24];
○ Extrinsic task arises from inconsistent lighting, environmental distractions, the use of the wrong shade determination tools, and the need to combine information from different sources [25,26,27].

**Figure 1:**
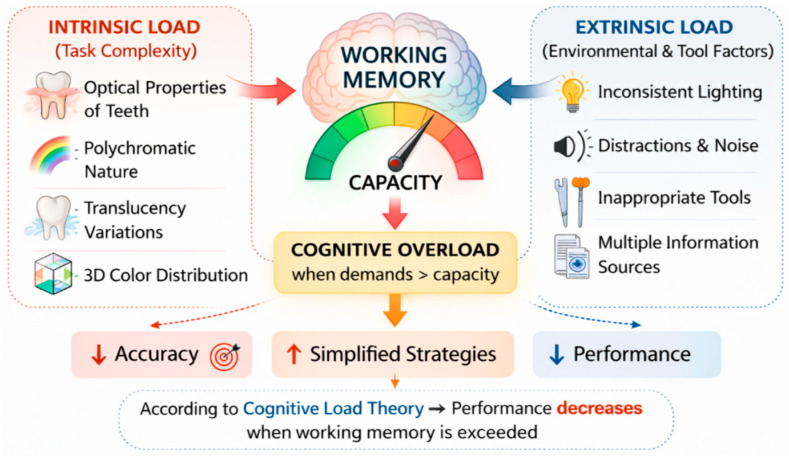
Cognitive Load in shade selection.

Translating Cognitive Load Theory into clinical practice offers some concrete recommendations. First, intrinsic load can be reduced by structured assessment protocols that break down the hue selection task into manageable, sequential steps. We can discuss the evaluation of value, followed by chroma, and last but not least hue. This intrinsic load focuses on a simultaneous multidimensional judgment [28]. Second, extrinsic load is minimized by standardizing lighting conditions (CIE D55/D65 illuminants), eliminating distractions, and using hue guides with logical spatial arrangements. Third, productive cognitive effort that promotes learning is enhanced by deliberate practice with immediate feedback, such as comparing visual selections with spectrophotometer readings to calibrate perceptual schemas. Clinics and educational institutions should design workflows and explicit curricula around these load management principles (Figure 1).

When cognitive load is too high, perceptual discrimination decreases, systematic evaluation decreases, and error rates increase. All of these are patterns observed in novice clinicians [29,30]. Imbery et al. evaluated the determinants of dental shade matching among dental students and found that certain tests that predict shade matching proficiency were not fully validated, indicating the multifactorial nature of this cognitive ability [21].

The environment in which color is captured continues to be a significant source of perceptual bias. Standard lighting in dental offices often has high color temperatures and uneven spectral power distributions, making it impossible to accurately match shades to standard daylight [31,32]. This causes uncertainty because a restoration that looks good in office lighting may not look good in daylight or other types of light.

Recent evidence indicates that even sophisticated intraoral scanners are susceptible to these fluctuations. In 2019, Reyes et al. showed that intraoral scanner-based shade registrations change significantly with lighting, and other researchers have shown that the accuracy and repeatability of digital shade matching algorithms are also affected by room lighting [17,33]. The transition to daylight-calibrated lighting sources in the operating room has demonstrated a reduction in variability. However, their implementation continues to present challenges for many clinicians [34].

Jouhar et al. directly compared students’ ability to match shades under fluorescent and corrective lighting. They found that all groups of students performed significantly better under corrective lighting. This finding has immediate implications for clinical practice, as improving operating room lighting may represent one of the most cost-effective strategies to reduce perceived difficulty and improve outcomes [31].

In addition to lighting, physical factors such as limited mouth opening, saliva-induced gloss, and patient movement create a high-pressure environment that complicates the clinician’s work and impairs their professional judgment [35].

### 3.2. Biological Dynamics

The dynamic nature of the oral cavity substrate introduces a temporal dimension to these difficulties. Once a tooth is isolated, either with a rubber dam or by prolonged lip protraction, it begins to dehydrate, leading to a significant increase in value, such as brightness, and a concomitant decrease in chroma, such as saturation [36,37].

Quantitative studies have characterized this dehydration effect. Within 5–10 min of isolation and air drying, the tooth brightness increases by approximately 2–3 CIELAB units (ΔL* = 2.1–3.4), while the tooth structure saturation decreases by 1.5–2.5 units (ΔC* = −1.6 to −2.4), corresponding to a clinically significant ΔE of 3.5–5.0 [38,39]. This exceeds the 50:50% acceptability threshold (ΔE < 2.7) and approaches or exceeds the 100% perceptibility threshold (ΔE < 1.2), meaning that the dehydrated tooth appears substantially lighter and less saturated than in its hydrated form. Rehydration with water for 2 min partially reverses these changes (approximately 60–70% recovery), but full recovery to the initial state requires 10–15 min of intraoral rehydration. A shade selected ten minutes after starting a procedure will differ from the initial appearance of the tooth, which significantly complicates visual shade determination.

The perceived difficulty stems from the clinician’s need to recreate the natural shade from a dehydrated substrate, involving a high cognitive load and often resulting in overcontoured or overly opaque ceramic prescriptions. Understanding the temporal evolution of dehydration and developing strategies to mitigate its effects, such as rapid assessment or rehydration protocols, is an important clinical skill that is rarely understood.

Furthermore, the influence of gingival architecture and the background effect of the oral cavity significantly alter the perception of translucency. Clinicians often have difficulty communicating these polychromatic shades to the laboratory, as single-point shade measurement fails to capture the dynamic interaction between the restoration and its biological environment [40,41]. Environmental instability, especially the lack of daylight-calibrated lighting, further exacerbates these biological changes.

### 3.3. Communication and Laboratory Synergy

An often-overlooked contextual factor is the information modification that occurs during data transfer from the clinic to the dental laboratory. Despite the promise of digital workflows, the lack of standardized communication protocols often leads to subjective interpretations of shades, such as incisal translucency and surface texture [42].

Esthetic dissatisfaction also stems from insufficient data transmission to the dental laboratory [6,43,44]. Recording a single shade or obtaining a single digital result, without being accompanied by additional data (such as polarized photography or detailed mapping), creates a communication gap that must be reworked. Ambiguity regarding the laboratory’s ability to interpret the clinician’s intent, especially for novice practitioners who lack the ability to communicate details to technicians, significantly influences the esthetic outcome [45].

In 2025, Konishi et al. demonstrated that dental technicians exhibit higher discrimination capacity and stricter acceptance criteria for lightness and chroma compared to dentists and patients [46]. This finding has profound implications for communication between clinician and technician, as if the two parties have different interpretations and acceptance thresholds, misunderstandings are inevitable. Standardized communication protocols, calibrated digital photography, and common reference points become essential to bridge this gap.

A concrete example illustrates this communication problem. A clinician visually selects “VITA 3D-Master 2M2” for a maxillary central incisor, but does not convey the information about the chroma in the cervical third, which is larger (3M2). The technician, receiving only “2M2”, designs a uniform 2M2 restoration. Upon seating, the patient rejects the restoration as “too flat” and “inappropriate”. Subsequent restoration requires three additional appointments, laboratory fees double, and patient satisfaction can be affected. This scenario, frequently reported in retrospective laboratory audits [47], exemplifies the economic and relational costs of communication deficits.

### 3.4. Patient Specific Factors

**Figure 2:**
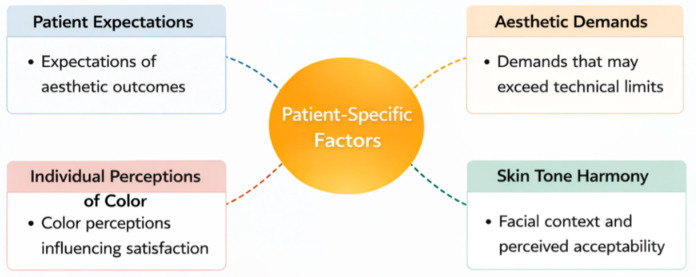
Patient Specific Factors.

Patient-related variables introduce additional levels of complexity beyond dental optics. Clinicians must manage:

- Patients frequently arrive for treatment with specific images of an ideal smile, which may not take into account the optical constraints of dental materials or their distinct oral anatomy. Therefore, the first consultation should include a lengthy discussion to ensure that the patient’s wishes match what is possible in the clinic. This will build trust and provide the patient with informed consent [48].
- A major clinical problem arises when a patient desires a perfect match, such as achieving the brilliance of a discolored shade on a single restoration, but the properties of the porcelain or composite make this impossible. In these situations, the clinician must tactfully explain these technical limitations while seeking other ways to treat the patient that will lead to the best possible outcome [49].
- Patient subjectivity in color perception varies, as each patient perceives and prioritizes color attributes such as hue, chroma, and value based on their own established cues. Clinicians must understand and manage these differences throughout the restorative process [50].
- External factors such as overall facial features, skin tone, and lip color contribute to color determination. Recent evidence confirms that skin tone significantly affects tooth shade selection and ignoring this broader facial context can lead to restorations that appear unsightly, despite alignment with an adjacent tooth shade [15,51] (Figure 2).

Studies indicate that patient satisfaction is affected by both objective shades matching and subjective esthetics and individual preferences. Evidence-based communication strategies, including engaging patients in shade discussions, using visual materials, clarifying material limitations, and addressing the impact of lighting, can alleviate dissatisfaction [52,53].

## 4. The Technological Dimension: Aid or Crutch?

### 4.1. Spectrophotometers and the Precision Interpretation Paradox

Many studies show that devices, such as the Vita Easyshade, are more accurate than traditional visual methods for determining shade [54,55]. Spectrophotometers measure color in standardized color spaces (CIELAB, CIELCH), which eliminates the variability that comes from how people see things. However, they are not yet widely used in clinics. Clinicians often report difficulties associated with:

- Positioning the probe on curved tooth surfaces, where edge loss effects can distort the information obtained [56].
- Controlling humidity, as saliva or dryness alter the appearance of things [54].
- Converting numerical results, such as L*a*b* or LCh coordinates, into easily understandable decisions that aid restoration [57,58].
- Inter-device variability, where identical units produce significantly disparate shade results despite high repeatability [18].

These problems are compounded by the fact that different devices have their own issues, such as environmental sensitivity and the fact that colorimetric data are abstract. Analysis paralysis is a term that describes what happens when there is too much data for the practitioner to manage, making them less likely to use them regularly. When instrumental results require additional cognitive processing to be clinically actionable, the anticipated improvement in difficulty may not occur.

The effectiveness of spectrophotometric shade selection is significantly correlated with the level of operator training. Alshiddi et al. showed that untrained dental students performed better with the Easyshade spectrophotometer than with visual methods [55]. However, trained students performed better with visual methods, particularly when it came to matching values (brightness). This contrast suggests that spectrophotometers may function as compensatory tools for novices, while experienced clinicians perceive visual methods as more intuitive and contextually flexible. Similarly, Parameswaran et al. found that although spectrophotometers had better inter-elevator agreement, they were less accurate than visual matching when used without positioning devices or calibration protocols [54].

Klotz et al. and Hampé-Kautz et al. found that the Easyshade V and Rayplicker devices had very good intra-device repeatability (ICC > 0.9) [57,58]. This supports their potential reliability under standardized conditions. However, even among trained individuals, the lack of a direct clinical correlation between ΔE values and perceptual thresholds can hinder decision-making. In 2018, Kim et al. observed that two identical Easyshade V devices produced significantly different shade results, despite high repeatability [56]. This discrepancy raises concerns about consistency between devices, thereby diminishing clinician confidence. These results highlight a major conflict. Although spectrophotometers provide objective data, the best way to use them in clinical settings depends on how skilled the user is, how well the device is designed for comfort, and how well the interface links numerical accuracy to visual perception. Technology meant to make things easier can actually make things harder if these supporting elements are not present.

### 4.2. Intraoral Scanners and Digital Workflows: The Redistribution of Cognitive Load

A critical distinction must be made between random error (imprecision) and systematic bias (inaccuracy) in digital shade matching devices. Random error refers to the variability of repeated measurements of the same tooth under identical conditions, quantified by repeatability coefficients or intraclass correlation coefficients. Systematic bias refers to the consistent deviation from the true shade value, usually assessed by comparing the device output to a reference spectrophotometer under standardized conditions. The literature indicates that modern devices generally exhibit an acceptable random error (ICC > 0.85 for most state-of-the-art spectrophotometers and scanners), which means that they are accurate. However, systematic bias is more problematic. Some devices consistently overestimate the value or shift the shade in specific directions, especially on discolored substrates or in non-central positions [56]. This distinction has clinical implications. Random error can be reduced by repeated measurements and averaging, while systematic bias requires device-specific calibration or algorithm adjustments.

Although intraoral scanners promise a simplified, one-click approach to shade selection, the literature consistently shows that digital shade matching introduces its own multi-layered challenges. Rather than eliminating the difficulty, scanners redistribute it from direct perceptual judgment to the cognitive, procedural, and environmental demands of digital workflows.

In several investigations, intraoral scanners demonstrate high repeatability but inconsistent accuracy, with performance strongly influenced by:

- Scanning technique and operator experience;
- Ambient lighting conditions;
- Algorithms used to convert captured images into shade guide values [40,59,60].

TRIOS-based systems generally achieve repeatability exceeding 85%, but have clinically variable accuracy, ranging from 22% to 66%, depending on the shade guide and reference instrument used [59,61,62]. A recent systematic review and meta-analysis by in 2024, Delija Omazić et al. compared intraoral scanners and spectrophotometers [10]. They found that intraoral scanners are not as accurate as spectrophotometers in terms of color matching, but are still considered accurate and similar to spectrophotometers. The combined ratio for precision was between 0.28 (CI: 0.09–0.60) and 0.38 (CI: 0.24–0.53), and the combined ratio for repeatability was between 0.81 (CI: 0.64–0.91) and 0.85 (CI: 0.74–0.92).

Environmental factors further complicate scanner performance. Although spectrophotometers are largely insensitive to variations in illumination, scanner-based color results fluctuate significantly with illumination. Reyes et al. reported paradoxically improved consistency in low-light or near-dark conditions, a counterintuitive finding that highlights the complex interplay between ambient light and scanner algorithms [33]. In 2025, Guo et al. similarly demonstrated that ambient lighting conditions have a significant impact on the accuracy and repeatability of digital shade matching algorithms [5].

A recent comparative evaluation by Hein et al. evaluated four intraoral scanners and a spectrophotometer for percentage correct shade identification in clinical dentistry [63]. The overall clinical success rate was highest for Carestream (78.2%), followed by Easyshade (63.5%), Primescan (51.2%), Trios (39.5%), and Medit (31.3%), with significant differences between devices observed for all categories (*p* < 0.05). These findings highlight substantial variability between devices and emphasize the importance of considering device performance when relying on digital systems for shade selection without visual verification.

These findings shift the perceived difficulty of shade matching from the clinician’s visual system to the clinician’s ability to navigate a digital ecosystem. Mastery now requires:

- Understanding scanner calibration protocols;
- Controlling environmental variables;
- Interpreting software-generated shade maps;
- Determining when to trust—or ignore—the device output.

As several authors have reported, clinicians often face uncertainty not because scanners are inherently complex, but because their algorithms hide how shade values are derived, creating a new level of cognitive load and professional hesitation [33,40]. In this sense, digital shade matching becomes a cognitive skill that requires not only technical proficiency but also metacognitive awareness of device limitations, contextual judgment, and the ability to integrate digital data with clinical intuition.

### 4.3. The Technology Confidence Gap

The technology trust gap reflects a nuanced paradox in contemporary shade selection, even though digital devices are intended to reduce uncertainty.

Their inconsistent performance may inadvertently influence clinicians’ perceptions of difficulty. Numerous studies have shown that spectrophotometers, colorimeters, and intraoral scanners exhibit variability even when using identical units under standardized conditions [42,63,64].

When the results of digital methods differ from a clinician’s visual assessment, the resulting cognitive dissonance can undermine trust.

This phenomenon is particularly pronounced among students and early-career practitioners who have insufficient perceptual calibration and rely heavily on external validation [55,65]. Inadequate training in device use, lack of understanding of algorithmic constraints, and irregular calibration protocols lead to misinterpretation of technological mismatches as personal errors rather than expected device performance [66,67]. Lee et al. found only moderate agreement between scanners and spectrophotometers in 2020 (Kw = 0.498 for VITA 3D-Master), with considerably lower agreement between scanners and visual matching, highlighting the continuing inconsistency between modalities [16]. Systematic research confirms that digital shade matching systems demonstrate variable accuracy, affected by device design, calibration, and environmental factors [42,68].

### 4.4. Multimodal Integration: The Clinical Reality of Hybrid Approaches

Emerging artificial intelligence systems represent the next frontier in digital color matching. Recent proof-of-concept studies have used convolutional neural networks trained on thousands of clinical photographs to predict color guide values directly from smartphone images, with preliminary accuracy rates of 75–85% compared to spectrophotometer references [69,70]. Machine learning approaches have also been applied to predict the final restoration color from pre-cementation trial shades, accounting for the effects of cement color and thickness [71]. However, current artificial intelligence systems share limitations with conventional devices. They are trained on specific populations and lighting conditions, their decision boundaries are opaque, and they have not been prospectively validated in clinical trials. Furthermore, AI introduces new cognitive challenges as clinicians must calibrate confidence in algorithmic results without understanding their derivation. The principle articulated for conventional devices, such as technology as verification not replacement, applies equally to AI systems, which are best positioned as decision support tools that provide probabilistic recommendations rather than definitive answers. A critical observation emerging from clinical practice, but underrepresented in the literature, is that experienced clinicians rarely rely on a single modality for shade selection. Instead, they employ flexible, context-dependent hybrid strategies that integrate visual inspection, instrumental verification, and photographic documentation in sequences tailored to the complexity of the case. Parameswaran et al. [54] observed that although spectrophotometers demonstrated superior inter-operator agreement under controlled conditions, trained clinicians consistently reverted to visual methods when device readings conflicted with their perceptual judgment, effectively using the instrument as a second opinion rather than the final arbiter. Similarly, Hein et al. [6] reported that the highest overall clinical success rates were achieved not by exclusive reliance on a single device but by sequential triangulation, i.e., initial visual assessment, followed by instrumental verification and reconciliation of discrepancies through structured deliberation.

This multimodal approach offers several advantages. First, it mitigates the limitations of any single method. Visual assessment remains contextually sensitive but suffers from perceptual errors, while instrumentation offers repeatability but may lack ecological validity for complex polychromatic surfaces. Second, deliberate comparison of visual and digital outputs provides ongoing perceptual calibration, reinforcing accurate color discrimination through immediate feedback. Third, hybrid workflows retain clinical judgment as the final arbiter, positioning technology as an adjunct rather than a replacement. The calibration-centric protocol proposed in Section 6 operationalizes precisely this integrative philosophy, recognizing that the question is not “which method is superior?” but rather “how can the methods be optimally combined?”.

Device–human disagreements represent valuable learning opportunities rather than clinical failures. When a spectrophotometer reading differs from a clinician’s visual assessment, the discrepancy forces explicit consideration of potential sources of error: Is the lighting not standardized? Is the tooth dehydrated? Is the device probe positioned correctly? Is metamerism present? This cognitive inquiry, involving the deliberate reconciliation of conflicting data streams, promotes perceptual calibration and the development of metacognitive skills. Educational programs should therefore design exercises that deliberately create and then resolve such disagreements, using the device as a feedback mechanism rather than an answer key. Longitudinal studies demonstrate that students exposed to structured digital visual reconciliation protocols show accelerated improvement in independent visual assessment compared to those using either method alone [72].

## 5. The Interdependence of Knowledge, Environment, and Perception in Achieving Esthetic Precision

Shade selection needs to be redefined as a cognitive skill that is inherently environment-dependent and cannot be reduced to either pure visual perception or pure technology. The paradox of expertise highlights that even experienced practitioners have difficulty in automatically recognizing patterns when faced with atypical optical properties, such as complex porcelain systems [73,74,75]. The clinician can synthesize data with subjective visual intuition, taking into account individual physiological factors, such as ocular dominance and the specific requirements of each clinical situation [35,76].

The following dimensions do not operate in isolation but form an interconnected network of mutually reinforcing factors (Table 1).

Priority justification:

○ High—Solutions that are immediately actionable, low-cost, or strongly supported by evidence (e.g., standardized lighting, hybrid protocols, structured training).
○ Medium—Solutions requiring curriculum changes or longer implementation timelines (e.g., spiral curriculum).

The core of this process is explained by Cognitive Load Theory. A clinician’s perception of difficulty is a direct result of the mental effort required, which is compounded by a lack of trust in technology [55,77]. This distrust is often fueled by the simulation–clinical gap, the well-known discrepancy between how a procedure looks on a digital map and how it looks in the patient’s mouth [13,21]. When a digital tool shows something different from what the clinician sees with their own eyes, it creates a mental conflict. This conflict adds unnecessary mental work and, in the high-pressure field of cosmetic dentistry, can lead to erroneous decisions and undermine a clinician’s professional confidence [48,78].

Dentists often lack adequate training in color science, forcing them to rely on the judgment of the digital scanner without understanding its functionality [74,79]. However, these scanners can give different results if the lighting or environment is not appropriate [34,80]. The dentist’s trained eye cannot always correct the mistake when it happens. This creates a frustrating cycle, an unreliable reading causes the dentist to overthink and question every subsequent decision which only compounds the first mistake [29,81].

## 6. Bridging the Gap from Difficulty to Mastery

Responding to this proposal provides a fundamental framework for developing more effective educational strategies, refining clinical workflows, and guiding the development of assisted technologies. Ultimately, understanding why shade selection is difficult is the first and most important step toward making it more predictable and effective.

One proposal could be a calibration-centric clinical protocol that positions digital technology as a verification tool within a fundamentally human-driven shade matching workflow. This model recognizes that technology should not replace clinical judgment, but rather serve to enhance and calibrate it.

The clinical protocol centered on calibration is performed according to the following steps:

Step 1: Preoperative assessment (2 min). Under standardized lighting conditions calibrated to daylight (CIE D55/D65, 5500–6500 K, CRI > 90), the adjacent and contralateral teeth are assessed for base, chroma, shade, and surface characteristics. The operative field is photographed with polarized filters to eliminate reflection.

Step 2: Tooth preparation and hydration management. Initially, if a rubber dam isolates the tooth, the initial shade assessment should be performed within 60 s. This helps establish a baseline before dehydration affects the color. If the assessment takes longer, the tooth should be rehydrated for two minutes using a water-soaked gauze pad before recording the shade.

Step 3: Visual assessment. Using a VITA 3D-Master shade guide, first determine the shade lightness by comparing the middle third of the tooth with the shade samples, with the eyes partially closed, to desaturate the color perception. Second, determine the chroma (saturation), selecting the most intense shade match. Third, record the shade, identifying any color changes (yellowish, reddish, gray). All 3 aspects of the shade are determined separately to obtain a shade that is as close as possible to that of natural teeth.

Step 4: Digital verification. Using a spectrophotometer (e.g., VITA Easyshade) or an intraoral scanner with a validated shade matching algorithm, three consecutive measurements of the middle third are taken, repositioning the probe between measurements. Record the modal (most frequent) result and the range of variation.

Step 5: Compare the visual and digital results. If the agreement is within clinically acceptable limits (ΔE < 2.7 or the same VITA 3D-Master shade-chroma-value triplet), proceed with the selected shade. If there is disagreement, systematically investigate potential sources such as lighting conditions, tooth dehydration, probe positioning, or metamerism. Repeat the evaluation after identifying the identified problems. If disagreement persists, prioritize visual evaluation for overall esthetic integration.

Step 6: Documentation and communication to the laboratory. Submit the final shade selection together with: (a) clinical photograph, (b) shade mapping diagram indicating cervical/medial/incisal variations, (c) digital verification results, (d) notes on surface characterization (translucency, opalescence, hypocalcifications), and (e) reference to adjacent tooth shades.

Step 7: Sample verification. When scheduling a trial, the restoration is evaluated under multiple lighting conditions, with these aspects being examined before final cementation.

The protocol proceeds as follows:

- The clinician establishes an initial shade hypothesis based on systematic assessment of value, chroma, hue, and surface characterization. This step remains indispensable because visual assessment, despite its susceptibility to observer-related variables, continues to serve as the most contextually sensitive and clinically intuitive method [55,82]. Numerous studies confirm that visual shade selection, when performed under optimized lighting conditions and by trained observers, can achieve high levels of reliability and remains the reference point against which digital results are interpreted [62,83].
- Once the visual baseline is established, a digital device, whether an intraoral scanner, spectrophotometer, or calibrated photographic system, is used to verify or refine the clinician’s initial selection. This sequencing leverages the complementary strengths of human perceptual judgment and instrumental reproducibility.
- When differences exist between visual and digital assessments, the clinician carefully reconciles them, considering possible sources of error in both types of assessments and using feedback from the device to improve perceptual calibration.

This focus on calibration has a number of benefits:

- It maintains clinical judgment as the most important factor in decision making;
- Leverages the strengths of technology (repeatability, quantification) without giving up the right to interpretation;
- Provides rapid feedback that aids perceptual learning over time;
- Reduces cognitive dissonance by presenting inconsistencies as opportunities for calibration, rather than failures.

**Figure 3:**
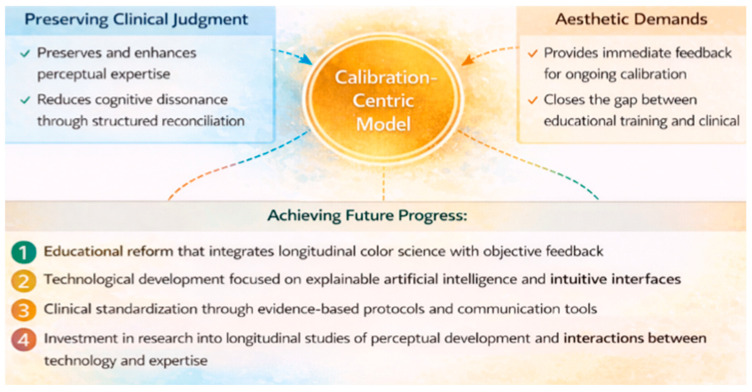
Specific Factors.

Figure 3 presents a logic diagram visualization of the calibration-centric hybrid protocol. The diagram illustrates sequential decision points, starting with the initial visual assessment, continuing with digital verification, and branching to the reconciliation stage. If there is agreement, the protocol proceeds directly to documentation. If there is disagreement, illumination, dehydration, positioning, and metamerism are investigated before either accepting the visual assessment (with digital documentation as context) or repeating the full protocol. The figure emphasizes that technology serves a verification function rather than an authoritative role, with clinical judgment retaining the final decision-making authority.

Research consistently shows that scanners and spectrophotometers have moderate agreement, while scanners and visual matching have even lower agreement. This suggests that we need hybrid approaches that combine the two rather than replace one with the other [26,84]. Studies indicate that training, repeated exposure, and structured feedback significantly improve shade matching accuracy among students and early-career practitioners [85,86,87] (Table 2).

## 7. Discussion

The literature contains apparent contradictions that warrant explicit discussion. First, while several studies report superior accuracy of spectrophotometers compared to visual methods [54,55], others find no significant difference or even superior visual performance among trained observers [62,83]. This contradiction is resolved when moderating variables are taken into account, such as whether the device superiority occurs under nonstandardized lighting conditions or with novice observers. Visual methods equal or exceed device performance under standardized conditions with experienced clinicians. Second, intraoral scanner performance ranges from 31% to 78% corrections across studies [6,10], a variability that is likely explained by device brand, ambient lighting, and operator technique. Third, studies reporting high repeatability (ICC > 0.9), reflecting the distinction between random error (low) and systematic bias (moderate to high) [56,57,58]. Rather than indicating methodological flaws, these contradictions reveal the context-dependent nature of shade-matching performance and caution against universal claims of device superiority.

The process of shade selection for indirect restorations represents a critical moment where art meets science and where the potential for error is high. This multidimensional difficulty is not attributable to a single factor but arises from the complex interplay between the observer (student or professional), the method (visual or instrumental), and the inherent limitations of the materials and systems themselves. Our synthesis of the recent literature confirms that while technology provides tools to augment human capacity, it does not eliminate the need for training and a deep understanding of color science.

A concrete clinical example illustrates the effect of expertise reversal. An experienced prosthodontist, who has successfully fitted thousands of single-shade crowns, is confronted with a maxillary central incisor with unusual internal features, such as a pronounced chameleon structure, incisal opalescence with a bluish halo, and cervical hypocalcification. The clinician’s established cognitive schema, namely, assessing value, then chroma, then shade, matching the closest guide sheet, fails because the tooth lacks a uniform shade. Under time pressure and high patient expectations, the expert reverts to slow, analytical processing, consciously considering each optical layer separately. Performance may temporarily fall below that of a senior resident who, without established schemas, applies a systematic polychromatic mapping protocol without experiencing cognitive conflict. This phenomenon, well documented in medical diagnosis [90], explains why experienced clinicians sometimes report greater difficulty with complex polychromatic cases than their less experienced colleagues.

Recent research by Konishi et al. investigated differences in tooth color discrimination and acceptance among dentists, dental technicians, patients, and dental students [46]. Significant factors influencing discrimination based on lightness were participant group and age, while those influencing acceptance were subject group and gender. Dental technicians demonstrated higher discrimination ability and stricter acceptance criteria for lightness and chroma, regardless of age. These findings suggest that professional role and training shape not only perceptual ability but also the evaluation standards applied during shade selection [91,92,93]. Expert clinicians typically rely on pattern recognition and internal visual framework developed through repeated exposure to diverse clinical cases. Studies show that experienced practitioners perform better than students in shade matching due to more refined perceptual categorization and faster intuitive judgments [94,95,96]. In 2024, Jouhar et al. demonstrated that clinical performance of dental students in shade matching improved with advancing years of dental education, confirming the developmental trajectory of this skill [8]. This expertise can deteriorate when cases fall into different patterns, such as teeth with atypical translucency, polychromatic, or complex internal characterization. Under these conditions, experts must revert to slower analytical reasoning, increasing cognitive load and perceived difficulty [97,98,99]. This phenomenon reflects the expertise reversal effect described in cognitive psychology, where expert performance declines when task complexity exceeds established schemas [100,101]. Therefore, difficulty in shade selection is not a challenge limited to beginners, it recurs whenever clinical complexity exceeds the clinician’s experiential framework, even for experienced practitioners who are faced with atypical optical properties or different ceramic systems [102,103,104].

A fundamental difficulty lies in the inherent subjectivity of human color perception. Students consistently demonstrate poor to moderate interrater agreement with traditional color guides, confirming that visual perception is a skill that requires deliberate development [105,106]. This challenge is not uniform, factors such as gender can influence discrimination ability, adding complexity to training and practice [107].

Human visual perception is further constrained by physiological limitations, color memory decay, and metamerism. Decision-making under conditions of perceptual uncertainty triggers cognitive heuristics that increase susceptibility to bias [108,109,110]. In addition, current dental programs often provide insufficient structured instruction in color science, creating a persistent gap between simulated learning and clinical requirements [111,112,113]. Environmental instability (variable lighting), biological dynamics (tooth dehydration), and communication gaps with laboratories all converge to challenge even experienced practitioners [114,115,116].

The instruments themselves contribute to the difficulty. Classical shade guides are poorly distributed in the natural tooth shade space, and the material difference between porcelain veneers and tooth structure affects light propagation, leading to metamerism and poor matches [117]. Remarkably, even shade guide labels alter clinicians’ shade preferences and accuracy, highlighting deep cognitive biases embedded in seemingly simple tasks [118]. Single-shade composite materials aim to circumvent this complexity, but systematic reviews confirm that their performance is strongly context-dependent, not providing a universal solution [119,120].

Instrumental methods such as spectrophotometers, intraoral scanners and smartphone applications have been heralded as the path to objective shade matching [121,122]. The literature claims that these devices offer greater repeatability and, under controlled conditions, greater accuracy than the average human observer [123,124,125]. Spectrophotometers such as VITA Easyshade are often considered reference standards [126,127]. However, a critical paradox arises, the difficulty does not disappear with technology, but rather moves into the technological and interpretative realm.

The accuracy of shade matching with intraoral scanner varies significantly between brands and even between units of the same brand [123,128]. Factors such as capture angle, ambient lighting and tooth surface characteristics such as moisture and tissue texture profoundly influence shade reading [129]. Precision, characterized by the ability to produce identical results on repeated measurements, is a separate concern, with studies demonstrating variable repeatability that undermines clinician confidence [130,131,132]. Comparisons of spectrophotometers reveal user-specific differences, suggesting that even objective instruments are not immune to operator or recording errors [133].

Studies comparing multiple devices frequently find disagreement, leaving clinicians uncertain about the use of a particular device [134,135,136]. Smartphone-based systems, while affordable, exhibit variable accuracy depending on hardware, algorithms, and lighting, positioning them as instruments with potential but significant limitations [137,138].

Direct comparisons reveal a nuanced picture. Although digital methods often outperform the naked eye in terms of raw accuracy [139,140,141], the margin is not always clinically overwhelming. A spectrophotometer can provide precise numerical data, but holistic assessment by an experienced clinician can produce a restoration that is more esthetically integrated with the surrounding dentition, taking into account translucency and surface gloss that colorimeters miss [142,143].

The choice of color difference formula (CIELAB vs. CIEDE2000) has a profound impact on whether the differences measured are clinically acceptable [144,145]. Perceptible and acceptable thresholds are not absolute, but are interpreted through mathematical formulas, influencing both research conclusions and clinical expectations.

For professionals and students, this creates a pedagogical paradox. Digital tools can guide beginners by providing objective feedback that calibrates developing visual senses [106,146]. However, overreliance on these tools without understanding their limitations is problematic. A student who accepts an intraoral scanner reading without questioning its validity in the case of a severely discolored tooth or a class V lesion is not approaching the clinical case esthetically correctly [147,148]. The real difficulty lies not in obtaining a number, but in interpreting its meaning [149]. Knowledge is gained not through technological substitution of judgment, but through calibrated integration, positioning digital tools as verification systems within fundamentally human-coordinated workflows [150,151].

The conceptualization of shade selection difficulty can be done on three levels:

- Perceptual Difficulty focuses on the human observer, influenced by training and experience [90,102,140]. This difficulty is greatest for beginners, but never disappears, as experts remain subject to fatigue, biases and limitations [118].
- Technological Difficulty arises from device limitations, due to instrument variability, the influence of uncontrolled clinical variables and the absence of a universal gold standard, generating contradictory data [11,123,134]. Users must master both color theory and the operational nuances of complex digital instruments [132,133,134].
- Educational Difficulty represents the meta-cognitive challenge of integrating perceptual and technological methods into coherent workflows. The curricula should encourage critical thinking about why a device gives a particular reading and how to communicate complex color information to technicians [91,132,142]. The goal is a synergy between digital vision and technology that augments, not replaces, but refines human judgment.

It is essential to recognize that the student–professional dichotomy, while useful, masks substantial variability within the group. Beginning students differ significantly from senior students, just as early-career practitioners differ from veteran clinicians. Therefore, this review synthesizes studies that use heterogeneous operational definitions of these categories, and the results should be interpreted as reflecting general developmental trends rather than fixed categorical differences.

The clinical reality of shade selection is inherently multimodal. Survey data indicate that most restorative dentists routinely use at least two methods, typically combining visual assessment with either spectrophotometric verification or standardized digital photography [88,89]. This approach reflects an intuitive recognition that no single modality is universally superior in all clinical settings. Evidence consistently demonstrates that method performance is strongly context-dependent, such as visual methods excelling under standardized lighting conditions for routine cases, spectrophotometers offering superior repeatability for single-tooth fits, and intraoral scanners offering workflow integration at the cost of reduced accuracy. Therefore, the optimal strategy is not strategic pluralism, namely selecting and sequencing methods based on case-specific factors, including restoration type, tooth position, adjacent restorations, and operator experience.

### 7.1. Limitations

This correlation of literature data is limited by patient-related factors, including enamel translucency, hydration status, and the optical properties of the underlying dentin or restorations. Studies that only analyze static components of dental tissues cannot fully demonstrate how complex they are as dynamic structures involved in light scattering. Furthermore, the variability of outcome measurements, ranging from spectrophotometric accuracy to subjective assessments, complicates definitive quantitative conclusions regarding the difficulty of shade determination between groups of observers.

Several limitations, beyond those mentioned above, warrant recognition. First, publication bias is likely present, as studies reporting positive or significant results (e.g., “Device X outperforms visual assessment”) are more likely to be published than those reporting null or negative results. Studies vary in terms of outcomes (pass/fail vs. ΔE values vs. agreement statistics), reference standards (different spectrophotometers, consensus panels, or no reference), lighting conditions, operator training, and dental substrates (natural vs. shades vs. restorations). This heterogeneity hinders meta-analysis and requires narrative synthesis. Third, patient-reported outcomes are conspicuously absent from the literature. Although numerous studies report device accuracy or operator agreement, very few measure patient satisfactions with shade match or the impact of mismatch on quality of life. The clinical significance of the observed ΔE differences remains uncertain without patient-centered validation. Fourth, most studies examine the shade match of a single tooth, while clinical reality frequently involves multiple restorations, adjacent discolored teeth, or posterior regions where access and illumination are compromised. Fifth, the predominance of cross-sectional designs precludes drawing conclusions about developmental trajectories or causal relationships between training and performance.

### 7.2. Future Perspectives

Research must move from documenting difficulties to validating solutions. Priorities include: (1) creating composite scoring systems that assess accuracy, cognitive load, and diagnostic reasoning; (2) longitudinal studies that map skill development from instruction to practice; (3) investigating digital devices as teaching tools that provide immediate feedback for perceptual calibration; (4) interdisciplinary collaboration with specialists using visual methods to isolate difficult optical dimensions; and (5) exploring artificial intelligence systems, augmenting expertise rather than replacing it.

## 8. Conclusions

Even after decades of advances in technology and new materials, achieving consistent results is still quite challenging. The color match between a restoration and natural teeth is perhaps the most visible indicator of clinical success or failure for both patient and clinician. Mismatched restorations have serious consequences including dissatisfied patients, costly rework (estimated $200–500 per remake), and profound professional frustration.

For the dentist, esthetic oral rehabilitation means developing the perceptual, cognitive, and technological skills necessary to successfully manage clinical complexity. As the field continues to evolve, the most appropriate approaches will be those that combine human judgment with technological precision. In today’s practice, methods must be tailored to the specific needs of each patient while maintaining the perceptual calibration that only deliberate practice can provide.

Due to the complexity of this topic, the conclusions can be structured according to the domain addressed. Regarding the clinical approach, it is recommended to adopt hybrid protocols centered on calibration (visual assessment first, digital verification second), implement a standardized operating lighting (CIE D55/D65), document shade selection with cross-polarized photographs and detailed characterization mapping, involve patients in shared decision-making regarding esthetic expectations.

In order to improve the educational system, it is recommended to integrate longitudinal color science into the curriculum, using spiral design, provide objective feedback through spectrophotometer verification exercises, develop competency-based assessments with explicit benchmarks, and reduce the gap between simulation and clinical through deliberate practice under varied lighting conditions.

The research domain can be approached by prioritizing longitudinal studies that aim to develop skills from novice to expert; validation of patient-reported outcome measurements for shade matching, investigation of AI systems as decision support rather than replacement; and establishment of consensus reference standards for device validation studies.

For device manufacturers, it can be concluded that interface design needs to be improved to reduce interpretive burden (e.g., visualizing ΔE differences rather than displaying abstract coordinates), providing explainable AI results, ensuring consistency across devices, and integrating calibration protocols into routine workflows. For the technician-physician team, establishment of standardized communication protocols, including shade mapping forms, cross-polarization photography, and regular calibration meetings, as well as recognition of different perceptual thresholds that require explicit negotiation, are recommended.

This synthesis highlights five priority areas for future investigation: (1) longitudinal cohort studies that map the development of shade matching skills from preclinical training to early clinical practice, identifying critical learning milestones; (2) randomized controlled trials that compare educational interventions (deliberate simulation-based practice vs. traditional training) on clinically meaningful outcomes (repeat rates, patient satisfaction); (3) validation studies of patient-reported outcome measures specific to shade matching, establishing thresholds for clinically important differences; (4) prospective evaluations of AI-assisted shade matching systems in real-world clinical settings, including assessing the effects of physician-AI interaction on decision quality and cognitive load; (5) implementation research that examines barriers and facilitators to the adoption of evidence-based shade matching protocols in diverse practice settings. As the field continues to evolve, the most appropriate approaches will be those that combine human judgment with technological precision, positioning technology as a tool for verification and calibration, rather than replacement. In contemporary practice, methods must be tailored to the specific needs of each patient, while maintaining the perceptual calibration that only deliberate and structured practice can provide.

## Figures and Tables

**Table 1:** Summary of the Four Dimensions of Shade Selection Difficulty.

Dimension	Key Factors Contributing to Difficulty	Primary Challenges	Evidence-Based Solutions	Priority Ranking
Technological	Spectrophotometer probe positioning Inter-device variability (31.3–78.2% pass rates) Black-box algorithms Ambient light sensitivity Edge-loss effects on curved surfaces	Paralysis by analysis Interpretive burden of numerical data Cognitive dissonance when device contradicts visual assessment Device-specific learning curves	Position devices as verification tools, not replacements Standardized calibration protocols User training in device limitations Integration with visual workflow	High
Cognitive–Psychological	Color memory decay (seconds) Retinal fatigue Metamerism under non-standardized lighting Physiological visual limitations Cognitive heuristics (anchoring, satisficing)	Perceptual instability Decision-making under uncertainty High intrinsic cognitive load Expertise reversal effect in complex cases	Structured visual assessment protocols Standardized lighting Cognitive load reduction strategies Deliberate practice with feedback	High
Educational	Insufficient hands-on practice Inadequate exposure to variable lighting Minimal digital tool integration Simulation to clinic gap Subjective, inconsistent feedback	Low student confidence Unrealistic training conditions Lack of perceptual calibration Delayed skill development	Longitudinal spiral curriculum Structured color science foundation Digital tools with objective feedback Deliberate practice under varied conditions	Medium
Clinical-Contextual	Non-standardized operatory lighting Tooth dehydration (value ↑, chroma ↓) Patient movement and saliva Soft-tissue reflectance Clinician-technician communication gaps	Environmental unpredictability Moving target of dehydrating teeth Information decay in laboratory communication Patient expectation management	Daylight-calibrated operatory lighting Rapid assessment protocols Cross-polarized photography Standardized communication protocols Patient involvement in shade selection	High

**Table 2:** Evidence Based Recommendations for Clinical Practice and Education.

Domain	Recommendation	Implementation Strategies	Expected Outcomes	Level of Evidence	Key References	Cost–Benefit Note
Clinical Environment	Implement daylight-calibrated operatory lighting (CIE D55/D65, CRI > 90)	Audit current lighting conditions Replace or supplement operatory lights Verify spectral power distribution Train staff on lighting importance	25–35% reduction in visual mismatch errors; ΔE improvement of 1.5–2.0 units under standardized lighting	I (systematic review)	Clary 2016 [25]; Jouhar 2024 [13]	Moderate cost ($500–1500 per operatory); rapid payback (estimated 5–10 remakes avoided)
Clinical Workflow	Adopt calibration-centric hybrid protocol (visual first, digital verification second)	Develop institutional protocol; Train clinicians; Provide devices for verification; Create documentation templates.	40–50% reduction in remakes; improvement in inter-operator agreement from κ = 0.4 to κ = 0.7	II (controlled trials)	Parameswaran 2016 [54]; Kim 2018 [56]	Low cost (training only); high benefit
Dehydration Management	Implement assessment protocols with rehydration strategies	Assess shade immediately after isolation Use rehydration techniques if delayed Document dehydration state with photos Communicate dehydration effects to laboratory	Maintains ΔE within 2.0 of hydrated baseline vs. 4.0+ without protocol	II (prospective studies)	Igiel 2017 [38]; Schmeling 2017 [39]	Negligible cost; immediate benefit
Clinician- Technician Communication	Standardize communication with calibrated photography and detailed mapping	Implement cross-polarized photography Use standardized shade mapping forms Include multiple views Document translucency	50–70% reduction in shade-related remakes; improvement in first-pass acceptance from 60% to 85%	II–III (laboratory surveys)	Konishi 2025 [46]; Hein 2024 [6]	Moderate cost (camera system $1000–3000); high benefit
Dental Education	Integrate color science longitudinally with spiral curriculum	Introduce basic concepts in preclinical years; Reinforce with clinical exercises; Integrate digital tools; Provide objective feedback	Improvement in student accuracy from 40–50% to 70–80% correct; sustained at 6-month follow-up	I–II (educational interventions)	Alshiddi 2015 [55]; Paravina 2019 [88]	Low-moderate cost; long-term benefit
Assessment in Education	Implement objective, standardized assessment tools with immediate feedback	Use spectrophotometers as reference standards Develop calibration exercises Create progressive benchmarks	Improvement in self-assessment accuracy (r = 0.35 to r = 0.65 correlation with expert assessment)	II (quasi-experimental)	Imbery 2022 [21]; Chimea 2020 [89]	Low-moderate cost; essential for competency
Technology Integration	Position AI and digital tools as decision support, not replacement	Train users on device limitations; Emphasize interpretive skills; Develop explainable AI interfaces;	Maintains clinical judgment while improving accuracy 10–15% over visual alone	III (expert consensus + preliminary studies)	Guo 2025 [5]; Morsy 2023 [69]	Variable; emerging evidence
Patient Communication	Involve patients in shade selection with visual aids and expectation management	Use shade comparison during consultation; Explain material and lighting limitations; Document patient preferences; Obtain informed consent	30–50% reduction in post-cementation dissatisfaction; improved satisfaction scores (1.5 point improvement on 10-point scale)	II–III (survey studies)	Alzeghaibi 2021 [49]; Sherif 2025 [48]	Negligible cost; substantial medicolegal benefit

Level of Evidence: I = systematic review/meta-analysis of RCTs; II = prospective controlled trial or high-quality cohort study; III = cross-sectional or lower-quality prospective study.

## Data Availability

No new data were created in this study.
